# Cross-validated multi-technique geodetic dataset of the Upper Adriatic Sea coastal area of Italy

**DOI:** 10.1016/j.dib.2022.108342

**Published:** 2022-06-01

**Authors:** Valeria Secreti, Marco Polcari, Letizia Anderlini, Matteo Albano, Mimmo Palano, Enrico Serpelloni, Salvatore Stramondo, Elisa Trasatti, Giuseppe Pezzo

**Affiliations:** a National Institute of Geophysics and Volcanology, National Earthquake Observatory, Via di Vigna Murata 605, I-00143 Rome, Italy; b National Institute of Geophysics and Volcanology, Bologna Section, Via Franceschini 31, I-40128 Bologna, Italy; c National Institute of Geophysics and Volcanology, Catania Section, Piazza Roma 2, I-95125 Catania, Italy

**Keywords:** Remote sensing, SAR Interferometry, GNSS, Levelling, Geodetic data, Ravenna coastal area

## Abstract

The geodetic dataset used in the research article entitled “*Multi-technique geodetic detection of onshore and offshore subsidence along the Upper Adriatic Sea coasts”*[1] is presented here. It consists of the outcomes of three different techniques, i.e. Synthetic Aperture Radar Interferometry (InSAR), Global Navigation Satellite System (GNSS) and topographic Levelling surveys. This dataset has been used for the estimation of onshore and offshore deformation in a mineral concession area located along the Upper Adriatic Sea coastal area (Italy), South-East of Ravenna city. InSAR data covers the period from 2002 to 2018, GNSS data from 1998 to 2018 and levelling data from 2002 to 2017.The different measurements have been cross-validated and referred to a common local reference system fixed in the urban area of Ravenna. This data collection will be very useful for deepening the analysis of any type of deformation in the Ravenna coastal area.

## Specification Table

**Table:** 

Subject	Earth and Planetary Sciences
Specific subject area	InSAR, GNSS and Levelling data
Type of data	Comma Separated Value (.csv) Shapefile
How data were acquired	InSAR data were retrieved from multi-temporal processing of Synthetic Aperture Radar (SAR) images acquired by Envisat and Sentinel-1 missions of the European Space Agency and by Cosmo-SkyMed missions of the Italian Space Agency. GNSS measurements were acquired by GNSS stations managed by public institutions and private companies. Levelling measurements were carried out through the use of benchmarks managed by ENI S.p.A.
Data format	Analysed
Parameters for data collection	InSAR, GNSS and Levelling data were collected to guarantee the best trade-off between temporal sampling, accuracy of measurements and spatial coverage of the Area of Interest (AOI)
Description of data collection	InSAR data provide ground velocity measurements (mm/yr) and displacement time series (mm) along satellite Line-of-Sight (LoS) direction for each point target in the AOI. GNSS measurements provide the North-South, East-West and Vertical components of the displacement rates (mm/yr) and position time series (mm) for each station in the AOI. Levelling surveys provide the vertical deformation velocity and displacement time series (m) for each benchmark inside the AOI.
Data source location	Ravenna coastal area, in the Northern Italy coastal areas. In particular the area around Lido di Dante and Fiumi Uniti. Upper left corner: 12.093° E; 44.489° N Lower right corner: 12.385° E; 44.225° N
Data accessibility	InSAR Repository name: INGV GeoSAR-IRiDIuM Archive InSAR Data identification number: https://doi.org/10.13127/insar/ts Direct URL to InSAR data: http://www.geosar-iridium.ct.ingv.it/landing/tmp/download.php?dir=/home/data/iridium/GeoSAR_INGV_Archive/InSAR_ground_displacement_time_series/Subsidence/Lido_di_Dante&folder=Lido_di_Dante GNSS and levelling Repository name: Zenodo GNSS and levelling Data identification number: https://doi.org/10.5281/zenodo.6563901 Direct URL to GNSS and levelling data: https://zenodo.org/record/6563901#.YoZV_qhBzIU
Related research article	M. Polcari, V. Secreti, L. Anderlini, M. Albano, M. Palano, E. Serpelloni, S. Stramondo, E. Trasatti, G. Pezzo, 2022. Multi-technique geodetic detection of onshore and offshore subsidence along the Upper Adriatic Sea coasts. Int. J. Appl. Earth Obs. and Geoinf. 108, 102756. https://doi.org/10.1016/j.jag.2022.102756

## Value of the Data

• The collected dataset is useful to improve the knowledge both in space and time of the ground displacement phenomena affecting the Upper Adriatic Sea coastal area, in fact the data cover a period of about 20 years (from 1998 to 2018).
• This dataset can help monitoring natural and anthropogenic phenomena for hazard assessment and risk reduction.
• The dataset can be used for deformation modelling studies in both inland and shoreline areas.
• The dataset can be reused and integrated with other data (such as ancillary ones) to have a synoptic view of the ongoing deformation in the Upper Adriatic Sea coastal area.
• The methodologies used to construct this dataset can be useful and provide a reasonable basis for similar work.
• These data can be useful to the scientific community for possible comparisons with other areas of the planet affected by the same phenomena.
• These data can be useful to geologists, geophysicists and engineers.

## 1 Data Description

**Fig. 1:**
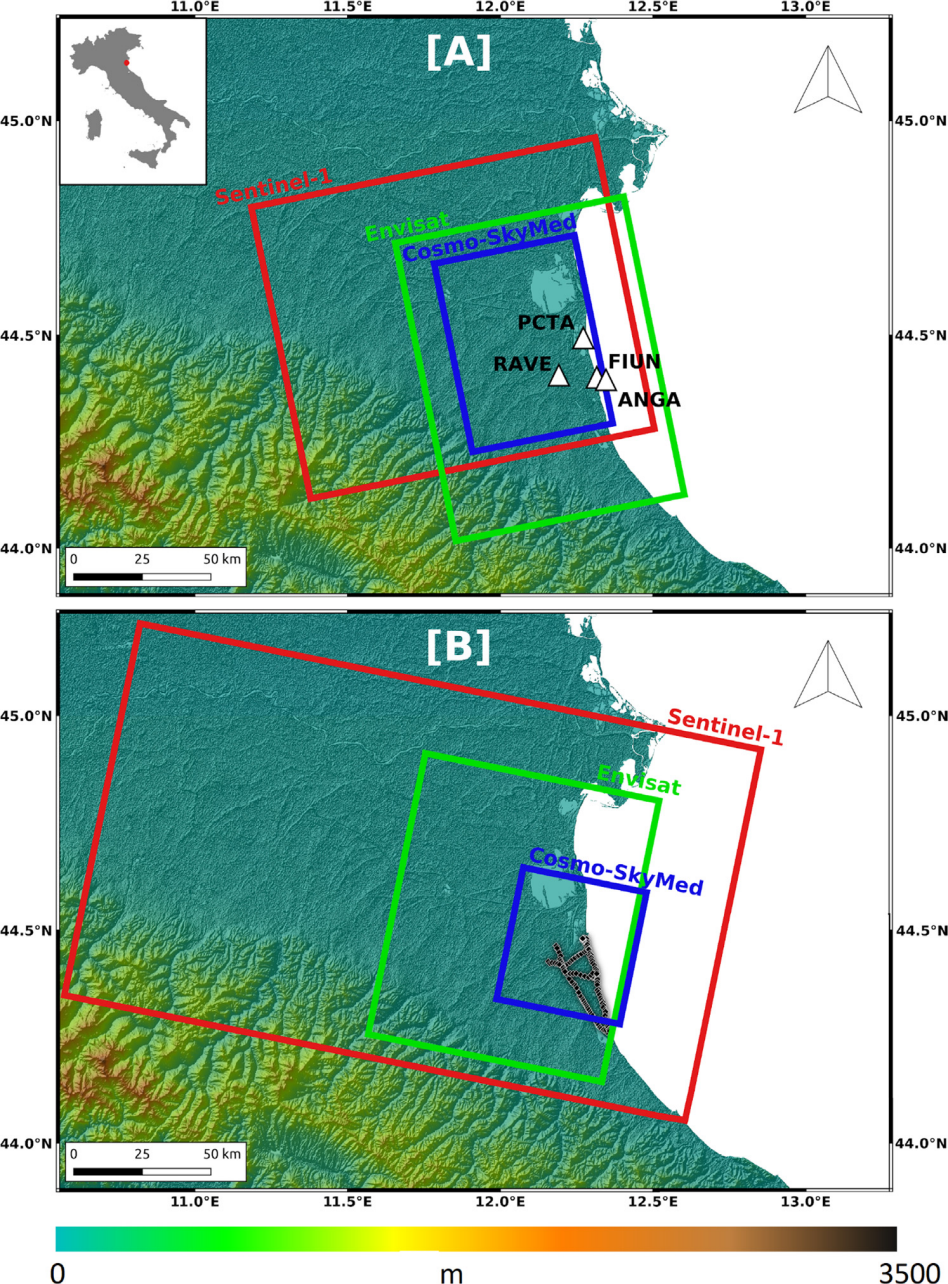
Focus on the dataset. Rectangles indicate the used Envisat, CSK and S1 SAR frames along ascending (A) and descending (B) track. White triangles are the GNSS stations whereas the black diamonds represent the levelling survey. The background image is the DEM provided by the SRTM mission.

This paper reports the geodetic dataset used to analyse the land subsidence in the Ravenna coastal area in the paper entitled “*Multi-technique geodetic detection of onshore and offshore subsidence along the Upper Adriatic Sea coasts”* [1]. It consists of Interferometric Synthetic Aperture Radar (InSAR), Global Navigation Satellite System (GNSS) and topographic Levelling data (Table 1 and Fig. 1).

**Table 1:** Number of points and time span of the data.

The remote sensing dataset is produced by multi-temporal InSAR processing of SAR images acquired along both ascending and descending tracks from Envisat, Cosmo-SkyMed (CSK) and Sentinel-1 (S1) space missions of the European Space Agency (ESA) and the Italian Space Agency (ASI). The covered time interval spans from 2002 to 2018 thanks to the continuity guaranteed by the different missions with only about one year-long gap occurring between the last Envisat image in 2010 and the first CSK image in 2011. InSAR processing was performed using 101 C-band Envisat images, 216 X-band CSK images and 227 C-band S1 images. The geometries of view are different according to the type of orbit and mission. Regarding Envisat, the ascending and descending geometries of view are characterized by an incidence angle of ∼21° and ∼24° respectively, and an azimuth angle of about 14° for both of them. For CSK, the ascending and descending geometries of view have an incidence angle of ∼27° and 34° and azimuth angles of ∼11° and ∼13°, respectively. For S1, the ascending and descending geometries of view are characterized by the same incidence angle, i.e. ∼ 39°, and by the same azimuth angle of about 14°.

The outcomes obtained from the processing of Envisat, CSK and S1 SAR datasets are provided as shapefiles format, along both ascending and descending orbits. Such shapefiles consist of tables with several attributes concerning each point target found in the InSAR processing step based on coherence threshold criterion. The first two attributes identify the latitude and longitude coordinates, expressed in decimal degrees in the WGS84 reference system. Third column is the height of the point target. Fourth and Fifth columns represent the estimated mean velocity of point targets and associated error, both expressed in mm/yr. Then, the following fields, i.e. those formed by initial D followed by a date (for example D20150330), are the Line-of-Sight (LoS) displacement values retrieved by InSAR analysis at the time of any SAR acquisition of the dataset. The provided shapefiles are optimized to be used in the open QGIS environment being compatible with most of its libraries and plug-ins such as the one for InSAR time series analysis (i.e., PS Time Series Viewer). According to the scientific aim of the main research article [1], the InSAR measurements are referred to the GNSS station located in Ravenna city but, upon request, InSAR data scaled with respect to Adria-fixed reference frame can also be supplied.

The GNSS data reported in this work consist of long-term displacement rates and displacement time series of the four permanent stations located in the AOI and named RAVE, PCTA, FIUN and ANGA (Fig. 1), approximately from 1998 to 2018. These stations are managed by both private companies and public institutions. In particular, the RAVE station belongs to the local authority of the Emilia Romagna region, while the other three belong to the ENI S.p.A. company. In Table 2 are reported the geographic coordinates of the stations, the long-term displacement velocity aligned to the local Adria-fixed reference frame in the three components (East-West, North-South, vertical) with the corresponding uncertainties (see the following sections for more details). The GNSS displacement time series are in .csv format and the measurements are provided both with respect to the Adria-fixed reference frame and to one of the four station, i.e. RAVE station. The tables have the same format, that is: epoch (time) of the position in decimal year, East (E) and North (N) components of the position in mm, uncertainties of the East (Se) and North (Ne) components in mm and correlation between them (Ren), Vertical component (U) of the position and its uncertainty (Su) both in mm, Reu and Rnu are the correlation between the vertical component and the East and the North ones, respectively, while the last three columns report the name of the site and the geographical coordinates in decimal degrees (longitude and latitude) of the station.

**Table 2:** Latitude and longitude of GNSS stations, velocity components with respect to the local Adria-fixed reference frame and corresponding uncertainties.

Station	Longitude	Latitude	EW	NS	UP	σ EW	σ NS	σ UP
ANGA	12.3436	44.3910	0.303	1.944	-17.757	0.086	0.116	0.394
FIUN	12.3159	44.3973	7.003	-1.789	-13.968	0.132	0.096	0.384
PCTA	12.2671	44.4948	2.223	2.328	-7.408	0.054	0.076	0.369
RAVE	12.1919	44.4053	1.085	1.052	-3.327	0.077	0.092	0.507

**Fig. 2:**
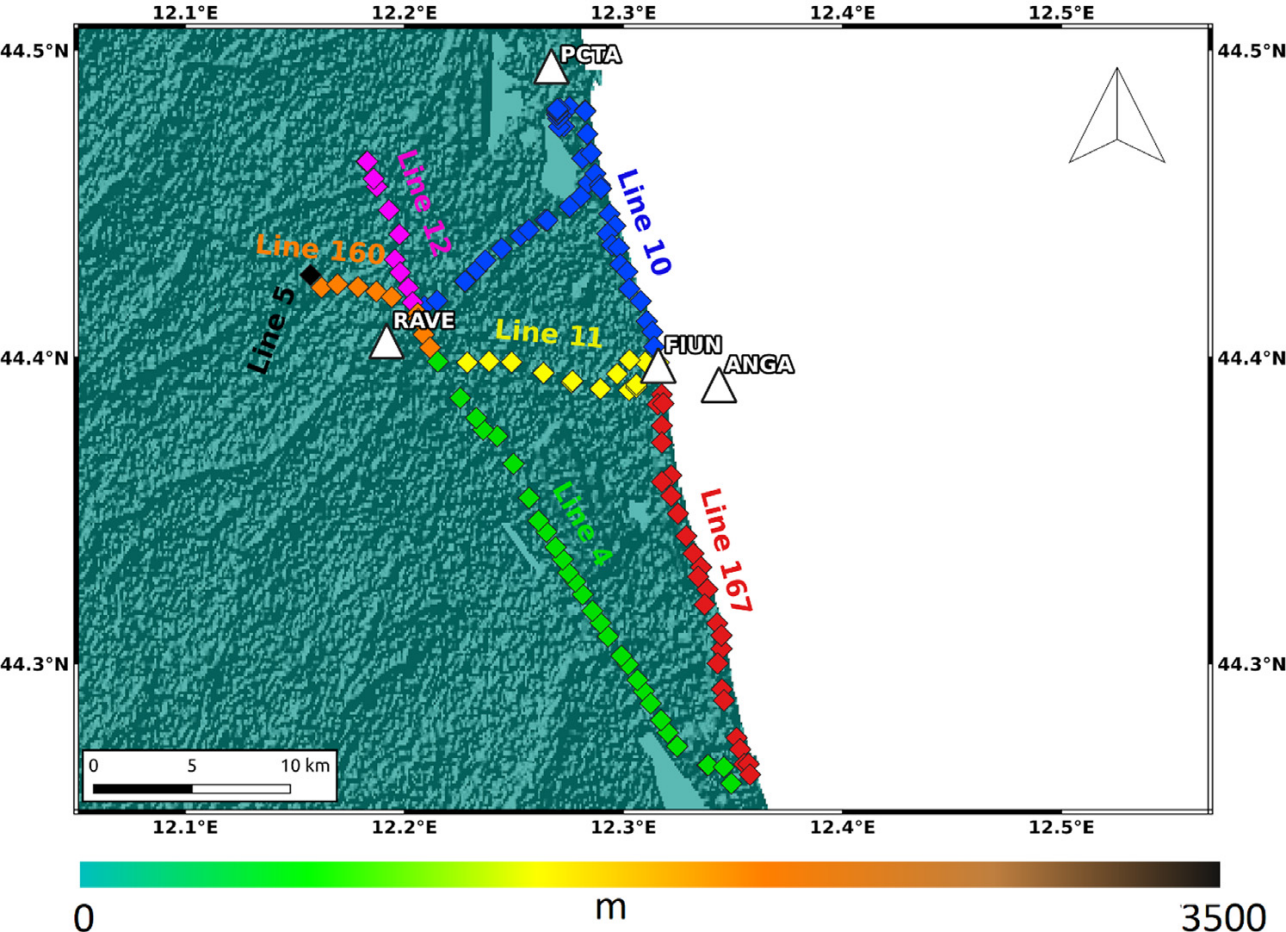
Location of GNSS stations and levelling benchmarks. The background image is the DEM provided by the SRTM mission.

The levelling data were provided by the Eni S.p.A. archives. The levelling surveys that took place in 2002, 2003, 2004, 2005, 2007, 2009, 2011, 2014, and 2017 were used, considering routes and lines across the AOI (4, 5, 10, 11, 12, 160 and 167), for a total of about 150 benchmarks (Fig. 2). Almost all the measurements made with the benchmarks, belonging to the lines 4, 10, 11, 12, 160, start from 2002 but there are some gaps within the time series. As for the benchmark belonging to line 5 (Fig. 2) instead, the campaigns carried out are: 2004, 2005, 2007, 2009. For line 167 almost all measurements start from 2003, also in this case there are some gaps in the data. Two files are provided, one with the original altitude measurements of the levelling benchmarks used and the other one with the displacement and the mean velocity calculated for each benchmark. Coordinates of each benchmark in the WGS84 reference system are also indicated.

The same reference area was chosen for all data, to cross-validate outcomes: the RAVE GNSS station for InSAR and GNSS data, a benchmark located near RAVE for the levelling data.

## 2 Experimental Design, Materials and Methods

### 2.1 InSAR data

The SAR data analysis was performed by the Multi-Temporal Interferometric Point Target Analysis (IPTA) approach, developed in the framework of GAMMA software [2]. Such approach merges the characteristics of the two main multi-temporal InSAR techniques, i.e. PS [3] and SBAS [4]. Indeed, on one hand it allows to build an interferograms network by appropriately setting the maximum thresholds for perpendicular and temporal baselines and, on the other hand, it works on full-resolution or averaged point targets to estimate the time series solution by Singular Value Decomposition (SVD) analysis.

Several processing steps were performed, selecting the parameters according to the features of each dataset. First, it was decided to work with pixel spacing of about 90 m since i) it is consistent with the scale of the phenomenon to be observed; ii) it is the same pixel size of the Digital Elevation Model (DEM) provided by the Shuttle Radar Topography Mission (SRTM) used for the removal of the topographic phase from the interferometric phase and iii) it allows to significantly reduce the speckle noise typical of SAR images.

Therefore, according to the original resolution of each dataset, multilook factors were applied along range and azimuth direction of 4 × 20, 30 × 30 and 24 × 6 for Envisat, CSK and S1 data, respectively.

**Fig. 3:**
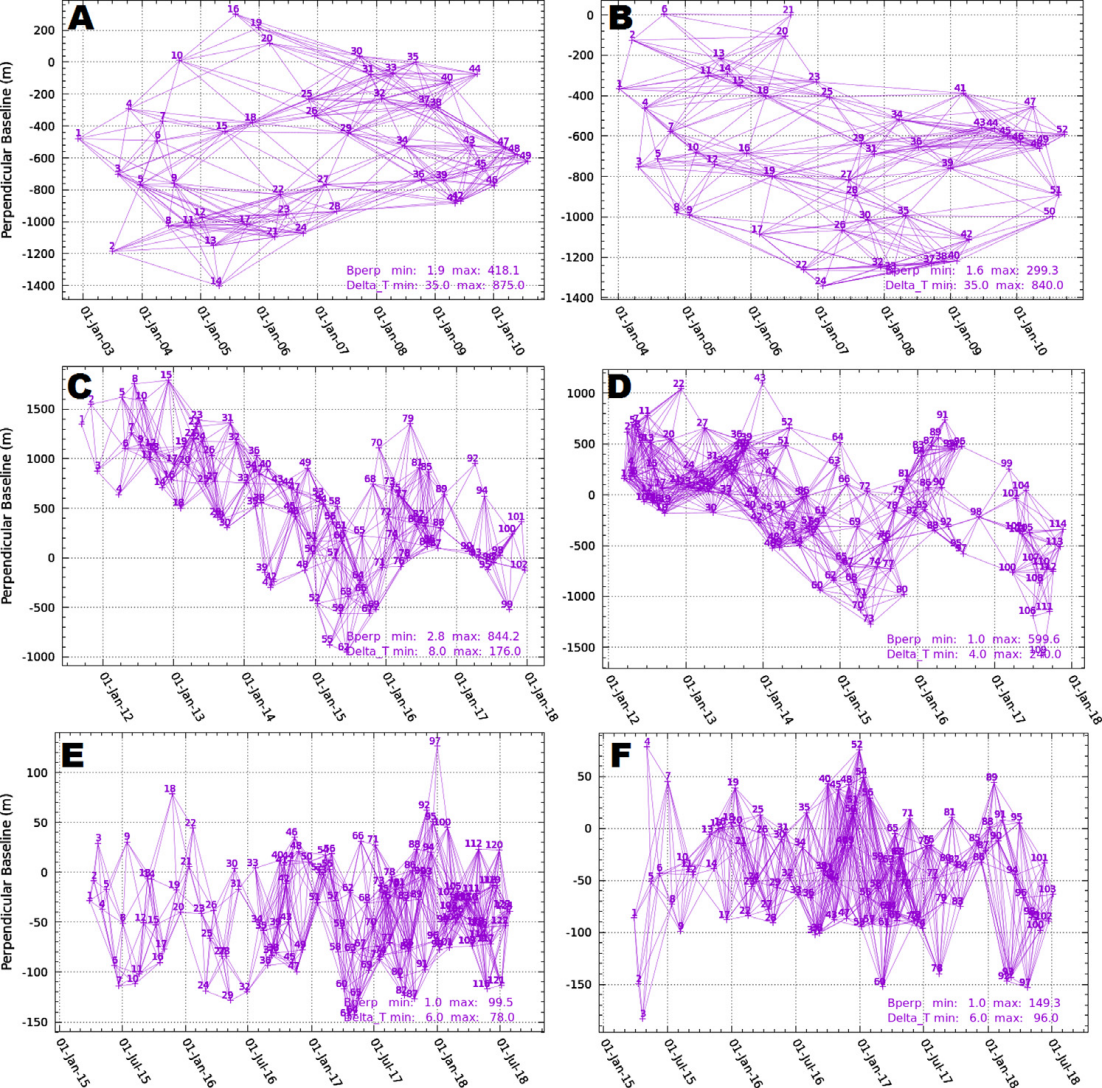
Interferograms network obtained for Envisat ascending (A) and descending (B); CSK ascending (C) and descending (D); S1 ascending (E) and descending (F).

With the aim of retrieving well connected interferograms networks, also the thresholds for the maximum perpendicular and temporal baseline have been set according to orbital tube and revisit time of the different missions. In particular, the interferometric pairs characterized by a maximum perpendicular and temporal baseline of 420 m and 900 days and 300 m and 850 days for Envisat ascending and descending data were selected. Concerning CSK data, such parameters were fixed at 850 m and 180 days for the ascending track and 600 m and 240 days for the descending one. Lastly, for S1 mission, characterized by the narrower orbital tube and the shorter revisit time, 100 m and 80 days and 150 m and 100 days were chosen, respectively, for the ascending and descending tracks. Such choices returned 259, 640 and 777 interferometric pairs for Envisat, CSK and S1 ascending data and 255, 917 and 776 for Envisat, CSK and S1 descending one (Fig. 3).

The differential interferograms were then filtered by Goldstein filtering [5] with an exponent parameter fixed to 0.8 and a window size equal to 32 pixels and unwrapped by minimum cost flow algorithm [6] using a coherence threshold of 0.4.

At this point, the selection of point targets was performed by coherence threshold criteria and the point target grids retrieved for each dataset have been used to sample all the estimated differential unwrapped interferograms. Finally, the computation of velocity rate and time series was obtained by SVD analysis. The final products are 6 shapefiles (one for each mission and each track, ascending and descending orbits) with latitude, longitude, deformation rate and error, along with the displacement time series for each point target.

### 2.2 GNSS data

The GNSS data reported in this work has been obtained analysing, on a daily base, the raw Global Positioning System (GPS) from more than 3000 continuous GNSS stations operating in the Euro-Mediterranean region (e.g. [7]) and maintained by public institutions and private companies. In this solution, the offshore GPS data provided by ENI S.p.A. [8] for the northern Adriatic Sea was also used. The analysis process of the raw GPS data uses the 10.7 version of the GAMIT/GLOBK software [9], adopting standards defined in the framework of the International GNSS Service (IGS) “Repro2 campaign” (http://acc.igs.org/reprocess2.html), and follows a three-step procedure, as described in Serpelloni et al. [7], which consists of: 1) raw phase data processing, 2) combination of daily loosely-constrained network solutions and reference frame definition and 3) displacement time series analysis, including velocity and seasonal signals estimates. In particular each step has been performed as follows:

1) The raw GPS observables (i.e phases of the recorded signals by the GNSS stations) have been analysed using the GAMIT package which allow to estimate loosely-constrained solutions in terms of station positions, atmospheric delays, satellite orbits, and Earth orientation parameters from ionosphere-free linear combination GPS double-frequency phase observables. This combination uses differencing techniques to eliminate phase biases caused by drifts in the satellite and receiver clock oscillators. GPS phase data are weighted according to an elevation-angle-dependent error model [9] using an iterative analysis procedure whereby the elevation dependence is determined from the observed scatter of phase residuals. In this analysis the satellites orbit parameters are fixed to the IGS final products. The IGS absolute antenna phase center model was used for both satellite and ground- based antennas, which improves the accuracy of estimates for the vertical components of site position by mitigating reference frame scale and atmospheric mapping function errors (e.g., [10]). While the first-order ionospheric delay is eliminated by the ionosphere-free linear combination, the second-order ionospheric corrections are applied based on the formulation of Petrie et al. [11], using IONEX files from the Center for Orbit Determination in Europe (CODE). The tropospheric delay is modelled as a piecewise linear model and estimated using the Vienna Mapping Function 1 (VMF1; [12]) with a 10° cutoff. The Global Pressure and Temperature 2 (GPT2; [13]) model was used to provide a priori hydrostatic delays. The pole tide was also corrected in GAMIT by the International Earth Rotation Service (IERS) standards. The Earth Orientation Parameters (EOP) are tightly constrained to priori values obtained from IERS Bulletin B. Non-tidal atmospheric loading and ocean tidal loading are corrected using MIT filtered atmospheric displacements files (available at ftp://everest.mit.edu/pub/GRIDS) and the FES2004 [14] model, respectively. The IERS 2003 model for diurnal and semi-diurnal solid Earth tides was set. Due to the large number of stations, the analysis has been performed into sub-networks with less than 50 stations, each of which shares a set of high-quality IGS stations, which are used as tie-stations in the combination step.
2) the daily loosely-constrained sub-network solutions are combined with the global solution of the IGS network made available by MIT (http://sopac.ucsd.edu) using the ST_FILTER program of the QOCA software (http://qoca.jpl.nasa.gov), which adopts a Kalman filter estimation algorithm (e.g. [15]). During this step the solutions are simultaneously aligned to a global reference frame minimizing the velocities of the IGS core stations (http://igscb.jpl.nasa.gov), while estimating a seven-parameter transformation with respect to the GPS realization of the ITRF2008 frame [16], i.e. the IGb08 reference frame.
3) the position time series have been modeled with a classic trajectory model [17] in order to simultaneously estimate offsets due to station equipment changes, annual and semi-annual periodic signals and a linear velocity term. Common Mode Error (CME) has been estimated and removed as in Serpelloni et al. [18] in order to improve the accuracy of the horizontal and vertical components.

The position time series, and thus the horizontal GPS velocities have been rotated in a local Adria-fixed reference frame using the rotation pole from Serpelloni et al. [18], in order to highlight more localized differential movements in the AOI. The corresponding velocities are reported in Table 2 and the displacement time series are provided in the dataset. In order to make all the datasets comparable to each other, GPS displacement time series referred to the RAVE station are also provided, while the GPS velocities can be easily extracted from Table 2.

### 2.3 Levelling data

ENI S.p.A. provided the height measurements obtained from the levelling campaigns. For any campaign, the altitude values were obtained through the least-squares adjustment according to the method of indirect observations. Starting from the altitude data, both vertical displacement and mean velocity were achieved for each benchmark taken into consideration. The first step focused on obtaining the vertical displacements through the following calculation:

$$H_{N}-H_{1}$$

where H_1_ is the height measured in the first year of the campaign while H_N_ represents the height measured in each subsequent year. In this way, the time series of vertical displacements for each benchmark were obtained. The second step focused on finding the mean velocities for each time series obtained. The mean velocity was calculated by linear regression analysis. This was done by use of the least-squares approach, through the best fit lines of the time series of each benchmark. In the end, in order to be able to compare and cross-validate the levelling data with both GNSS and InSAR data, the outcomes were scaled with respect to the mean velocity of the benchmark near the RAVE GNSS station.

## Ethics Statements

This work did not involve human and animal subjects neither did data from social media platform.

## Supplementary Information

The GNSS and levelling data are stored in the Zenodo repository [19], while InSAR data is in the INGV GeoSAR-IRiDIuM Archive [20].

## CRediT Author Statement

**Valeria Secreti:** Data curation, Methodology, Writing – original draft preparation; **Marco Polcari:** Conceptualization, Data curation, Methodology, Writing – original draft preparation; **Letizia Anderlini:** Data curation, Methodology, Writing – original draft preparation; **Matteo Albano:** Writing – review & editing; **Mimmo Palano:** Writing – review & editing; **Enrico Serpelloni:** Data curation, Writing – review & editing; **Salvatore Stramondo:** Writing – review & editing; **Elisa Trasatti:** Writing – review & editing; **Giuseppe Pezzo:** Project administration, Funding acquisition, Writing – review & editing.

## Declaration of Competing Interest

The authors declare that they have no known competing financial interests or personal relationships that could have appeared to influence the work reported in this paper.
